# Digital Cardiovascular Twins, AI Agents, and Sensor Data: A Narrative Review from System Architecture to Proactive Heart Health

**DOI:** 10.3390/s25175272

**Published:** 2025-08-24

**Authors:** Nurdaulet Tasmurzayev, Bibars Amangeldy, Baglan Imanbek, Zhanel Baigarayeva, Timur Imankulov, Gulmira Dikhanbayeva, Inzhu Amangeldi, Symbat Sharipova

**Affiliations:** 1 Faculty of Information Technology, Al-Farabi Kazakh National University, Almaty 050040, Kazakhstan; tasmurzayev.n@gmail.com (N.T.); zhanel.baigarayeva@gmail.com (Z.B.); imankulov.timur@gmail.com (T.I.); g.a.dikhanbayeva@outlook.com (G.D.); inzhu.sapargalikizi@gmail.com (I.A.); sharipovaa.simbat@gmail.com (S.S.); 2 LLP “DigitAlem”, Almaty 050042, Kazakhstan; 3 LLP “Kazakhstan R&D Solutions”, Almaty 050040, Kazakhstan

**Keywords:** digital cardiovascular twin, machine-learning, personalized medicine, generative AI, medical large-language models, AI agents, personalized intervention

## Abstract

Cardiovascular disease remains the world’s leading cause of mortality, yet everyday care still relies on episodic, symptom-driven interventions that detect ischemia, arrhythmias, and remodeling only after tissue damage has begun, limiting the effectiveness of therapy. A narrative review synthesized 183 studies published between 2016 and 2025 that were located through PubMed, MDPI, Scopus, IEEE Xplore, and Web of Science. This review examines CVD diagnostics using innovative technologies such as digital cardiovascular twins, which involve the collection of data from wearable IoT devices (electrocardiography (ECG), photoplethysmography (PPG), and mechanocardiography), clinical records, laboratory biomarkers, and genetic markers, as well as their integration with artificial intelligence (AI), including machine learning and deep learning, graph and transformer networks for interpreting multi-dimensional data streams and creating prognostic models, as well as generative AI, medical large language models (LLMs), and autonomous agents for decision support, personalized alerts, and treatment scenario modeling, and with cloud and edge computing for data processing. This multi-layered architecture enables the detection of silent pathologies long before clinical manifestations, transforming continuous observations into actionable recommendations and shifting cardiology from reactive treatment to predictive and preventive care. Evidence converges on four layers: sensors streaming multimodal clinical and environmental data; hybrid analytics that integrate hemodynamic models with deep-, graph- and transformer learning while Bayesian and Kalman filters manage uncertainty; decision support delivered by domain-tuned medical LLMs and autonomous agents; and prospective simulations that trial pacing or pharmacotherapy before bedside use, closing the prediction-intervention loop. This stack flags silent pathology weeks in advance and steers proactive personalized prevention. It also lays the groundwork for software-as-a-medical-device ecosystems and new regulatory guidance for trustworthy AI-enabled cardiovascular care.

## 1. Introduction

Reactive cardiology describes interventions that commence only after a cardiovascular event has been clinically confirmed. In this model, diagnosis and therapy are applied in response to an already-occurring myocardial infarction, arrhythmia, or heart-failure exacerbation rather than being used for early detection and disease prevention. Although such strategies have reduced acute mortality over decades, they remain constrained by late intervention, symptom dependency, and an inability to influence long-term cellular and molecular disease processes, which is especially evident amid the growing burden of cardiovascular disease in aging and high-risk populations [1].

Current research highlights the multifaceted limits of the reactive approach. Late detection of pathology restricts the ability to reverse progressive tissue damage, while fragmented care and insufficient integration of psychosocial and environmental risk factors aggravate the situation. Economic and technological barriers also impede effective patient management. Clinical trials demonstrate that treatment initiated after irreversible myocardial changes, arrhythmias, anthracycline cardiotoxicity, or inflammatory reactions in COVID-19 leads to poorer long-term outcomes [2,3]. It is especially critical that many diagnostic methods register damage only after they reach a clinically significant threshold. An increase in cardiac troponin indicates necrosis that has already occurred, and by the time the marker is detected, the area of ischemia often expands and the therapeutic window narrows [4]. In pediatric cardio-oncology, echocardiographic signs of anthracycline toxicity—left-ventricular dilatation and wall thinning—are identified at late stages of remodeling, when the chances of complete cardiac recovery diminish [5].

The concept of proactive healthcare marks a shift from reactive treatment to strategies focused on early diagnosis and timely intervention. This paradigm is particularly crucial for cardiovascular diseases, which are often detected only in later stages or after acute events, especially in patients with comorbid conditions that mask symptoms, such as type 2 diabetes and chronic obstructive pulmonary disease [6]. One of the most transformative aspects of proactive cardiology is the integration of digital technologies and wearable devices that deliver continuous real-time monitoring of cardiovascular parameters. Smartwatches, fitness trackers, and portable ECG monitors have revolutionized the field by enabling the collection of physiological data outside traditional clinical settings. These devices remotely track heart rate, blood pressure, and blood oxygen saturation, providing clinicians with a rich stream of information for early risk assessment and timely intervention. The uninterrupted flow of data makes it possible to detect even minor changes in a patient’s cardiovascular status, offering a clear advantage over the episodic in-office examinations typical of the reactive model [7].

Digital cardiovascular twin technology represents a paradigm shift in precision medicine, creating a virtual copy of the patient’s cardiovascular system that is continuously updated with real-time, multimodal data. The concept of digital twins originated in industry but quickly found applications in healthcare and particularly in cardiology [8].

Real-time data collection relies on wearable sensors that track heart rate, blood pressure, and electrocardiography [8]. Smartwatches and specialized ECG monitors generate continuous physiological streams that are transmitted wirelessly to cloud or edge devices for analysis [9]. This ecosystem provides not only constant monitoring but also instantaneous detection of arrhythmic patterns and ischemic events [10]. Internet-of-Things platforms enable seamless delivery of these data from the patient’s environment into the digital-twin system [8].

Medical imaging using MRI, CT, and echocardiography forms the anatomical foundation of the model. Updating this geometry is possible through periodic examinations, although continuous non-invasive imaging remains a challenge and a subject of active research [11]. High-fidelity heart models with sub-millimeter resolution require substantial computational resources [12]; therefore, reduced-order models and surrogate methods have been developed to approximate cardiovascular behavior at much lower computational cost while preserving critical physiological detail [11]. Digital twins, initially developed in engineering fields such as aerospace and manufacturing, have been extended to healthcare to create dynamic, continuously updated virtual copies of patients or specific organs [13]. In cardiovascular disease, a digital twin is a computational model that simulates the unique anatomical, physiological, and functional characteristics of a person’s heart and vascular system, integrating data from various sources collected in real time [14]. Real-time data collection is crucial for these models to reflect the patient’s current state and evolution, thereby supporting proactive, personalized treatment strategies and continuous monitoring for early deterioration detection [8].

The basic architecture of a digital cardiovascular twin consists of three main components: data acquisition, computational modeling, and real-time feedback integration. Data acquisition involves gathering various data types from multiple sources, including physiological sensors (e.g., continuous blood-pressure monitors, heart-rate sensors), imaging systems (MRI and CT scanners), and electronic health records (EHRs), which provide historical and clinical context [15]. Advanced wireless sensor networks and IoT devices play a key role in capturing continuous data streams that are relayed to cloud and edge computing infrastructures for immediate processing and analysis [11]. These sensors often use technologies such as Bluetooth, 5G, and other low-latency communication protocols to ensure seamless transmission of high-frequency biometric and environmental data [8].

Imaging data plays a crucial role in constructing the anatomical scaffold of the digitized heart, as patient-specific imaging techniques like CT and MRI provide detailed three-dimensional reconstructions required for modeling. After acquisition, these data streams are often pre-processed by algorithms that correct noise and missing values, using methods such as Bayesian denoising or moving-average filters to ensure data integrity and consistency [11]. The digital-twin paradigm combines mechanistic models with data-driven approaches to capture the complexities of cardiovascular physiology. Mechanistic models are based on established principles of physics and physiology, including equations describing blood-flow dynamics, myocardial contraction, and electrophysiological conduction [16]. Simultaneously, machine-learning models—including deep neural networks and graph-based predictive systems—process heterogeneous inputs, forecast future cardiac events, and adjust simulation parameters accordingly [17].

Real-time data acquisition is ensured by full sensor integration, advanced signal processing, and robust communication infrastructure. In modern digital-twin implementations, the data-collection infrastructure typically includes arrays of wearable sensors interconnected with cloud computing services that perform continuous data assimilation [8]. These sensors, whether embedded in smartwatches or integrated into hospital monitoring systems, provide virtually continuous time-stamped measurements that are transmitted to a central repository where they can be combined with historical clinical data [18]. Successful real-time data acquisition depends on the reliability and accuracy of the sensors themselves and on the design of the supporting communication networks. Many systems employ edge computing to reduce latency and offload primary data processing from central servers, allowing faster adjustments to the digital-twin model based on immediate physiological feedback [11]. This edge-to-cloud paradigm facilitates continuous model updates while balancing computational loads between local devices and remote data centers [19].

The digital cardiovascular twin greatly benefits from feedback mechanisms that allow it to update and simulate various scenarios in real time; for example, virtual interventions such as simulating pacing or drug administration can be tested in the digital environment before being applied to the patient [20]. Real-time data integration also supports dynamic risk stratification through continuous monitoring of biomarkers and vital clinical parameters that can signal an impending cardiovascular event [9]. Digital twins facilitate the planning and simulation of complex cardiac procedures, such as catheter ablation for atrial fibrillation or the optimization of implantable cardiac devices. By virtually testing different intervention scenarios on a patient-specific model, clinicians can estimate likely clinical outcomes and tailor their approach to maximize efficacy while minimizing side effects [16]. Employing such a continuously updated digital model transforms traditional reactive care into a more predictive and personalized therapeutic environment [21].

Machine-learning models are especially effective at extracting hidden patterns from the multidimensional data produced by continuous-monitoring devices, enabling the digital twin to adjust its forecasts in real time as new sensor inputs arrive [22]. Algorithms such as Bayesian estimation and Kalman filters further ease parameter tuning under uncertainty, ensuring the virtual replica remains an accurate reflection of the physical state [11]. This fusion of artificial intelligence and traditional modeling not only enhances simulation fidelity but also delivers clinicians actionable insights for real-time decision-making [8].

This narrative review introduces a hybrid AI–digital-twin design explicitly tailored to cardiovascular care: it couples beat-level ECG/PPG and motion streams with mechanistic hemodynamics via Bayesian/Kalman estimation to infer latent cardiovascular states; includes a cardiovascular scenario engine to simulate what-if effects of antihypertensives, rate/rhythm strategies for atrial fibrillation, and rehabilitation loads, with guardrails for guideline and drug–interaction safety; personalizes the twin using echocardiography indices, cardiac biomarkers, longitudinal rhythms and HRV to update model parameters and risk stratification; and tightly integrates medical LLMs trained on cardiology corpora to translate twin states into guideline-concordant, human-readable recommendations. Together, these elements go beyond generic digital-health twins by centering hemodynamics, rhythmology, and therapy titration that are specific to cardiology.

**Figure 1:**
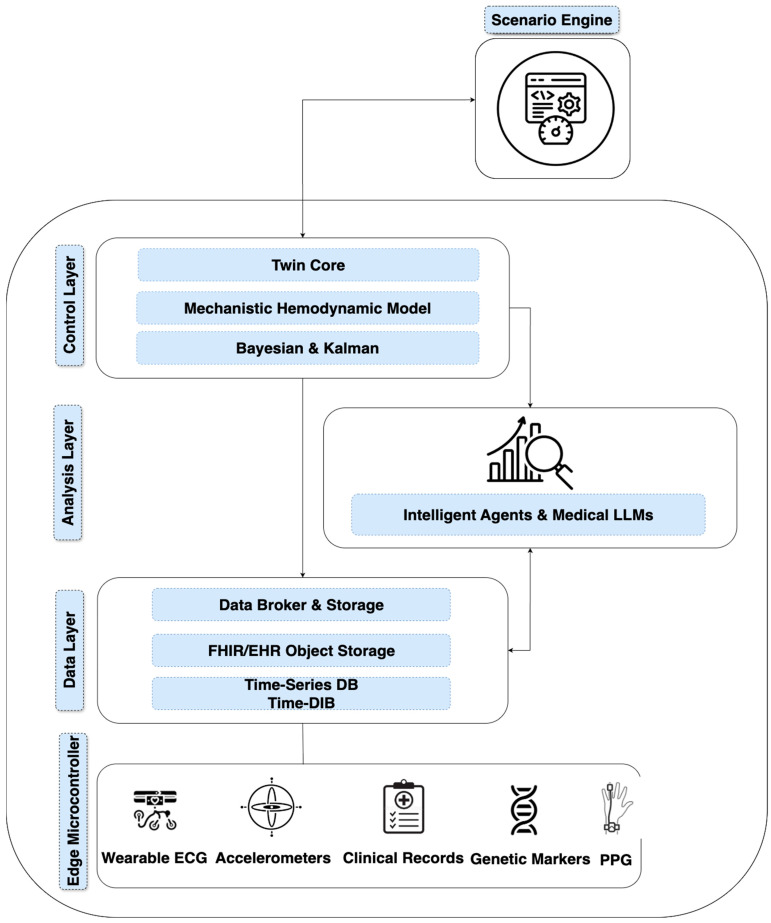
Four-layer cardiovascular digital twin: data ingestion/synchronization → cleaning/feature extraction → physics + ML twin for state/event forecasts → guideline-aligned recommendations with feedback recalibration.

The architecture of the cardiovascular digital twin, in Figure 1, is described through four interconnected layers. The data layer forms a multimodal stream. Wearable ECG and PPG devices, accelerometers, smart shirts, clinical records, and genetic markers are filtered and synchronized on edge microcontrollers before being forwarded to cloud or local repositories for further processing. Edge signals (wearable ECG/PPG, accelerometers, clinical records, genetic markers) enter the Data Layer via a broker, where they are timestamped, schema-validated (FHIR), and routed: raw waveforms to object storage, and aggregates/features to the time-series database. The Analysis Layer subscribes to the broker, extracts features, and produces predictions (risk scores, anomalies, therapy-response forecasts); the outputs are written back to the Data Layer as versioned artifacts. The Control Layer (Twin Core + mechanistic hemodynamics + Bayesian/Kalman) consumes both primary streams and analytics outputs, estimates state/parameters near real time, and generates decisions/alerts; these decisions are persisted in the Data Layer and sent to the Intelligent Agents & Medical LLMs block. The agent/LLM takes the current twin state and the analytics explanations to produce human-readable recommendations; clinician feedback is also logged in the Data Layer. The Scenario Engine operates in a ‘sandbox’ with the Control Layer: it receives the current twin state, runs what-if interventions, and returns expected trajectories; only approved scenarios proceed to execution.

## 2. Methodology

The objective of this review is to analyze the existing scientific literature to construct a comprehensive architecture for an AI-driven digital cardiovascular twin. Although this is a narrative review, we have employed a systematic approach to the search and selection of relevant studies to ensure methodological rigor and transparency. The process was divided into two key stages: (1) a literature search and screening in accordance with the PRISMA 2020 guidelines, and (2) a thematic analysis and classification of the selected works to structure the main body of the review.

To identify relevant publications, a search was conducted in leading scientific databases, including PubMed, IEEE Xplore, Scopus, and Google Scholar, using a combination of keywords (“digital cardiovascular twin”, “AI in cardiology”, “predictive cardiac model”, “wearable ECG/PPG analysis”).

**Figure 2:**
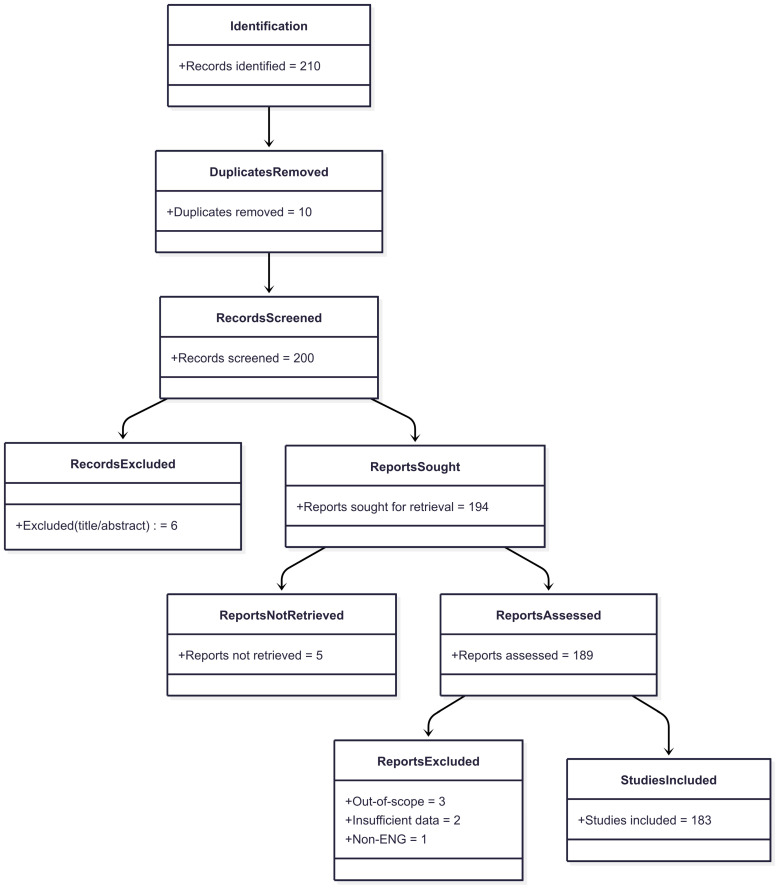
PRISMA flow diagram of study selection.

The study selection process is presented in the PRISMA diagram (Figure 2). The initial search identified 210 records. After removing duplicates (*n* = 10), a screening of titles and abstracts was conducted, which resulted in the exclusion of 6 works. For further analysis, 200 reports were selected, of which 6 could not be retrieved. The remaining 194 articles underwent a full-text assessment, after which 5 works were excluded for the following reasons: out of scope (*n* = 3), insufficient data (*n* = 2), and a publication language other than English (*n* = 1). Thus, the final sample for analysis included 183 studies. This structured approach ensures transparency in the formation of the literature corpus on which our analysis is based.

To ensure transparency in classification and to structure the review, each of the 183 publications was assigned to a dominant section based on its primary research objective (in cases of thematic overlap, only a single label was assigned). The distribution of articles across the sections is as follows:

Section 1 includes 22 papers that establish the conceptual foundation: the limits of reactive cardiology, the shift toward proactive medicine, definitions of the digital cardiovascular twin, and the role of real-time data streams.

Section 3 includes 63 publications focusing on the sensing/data layer (wearables, ECG/PPG, specialized medical sensors, EHRs) and the algorithmic layer—diagnostic deep-learning models for ECG/echocardiogram/imaging analysis, predictive models of CVD risk, and their personalization.

The largest section, Section 4, contains 59 articles on generative technologies, medical LLMs, and proactive AI agents for continuous monitoring, intelligent alerts, and recommendations.

Finally, 39 papers are included in Section 5, which analyzes issues of data source interoperability, sensor reliability and accuracy, computational complexity, clinical and regulatory validation (FDA/EMA), as well as challenges related to privacy, bias, and cybersecurity.

## 3. Components of the Digital Cardiovascular Twin

### 3.1. Sensory Layer

The sensory layer consolidates four complementary data domains—physiological, clinical, molecular, and environmental—into one continuous multimodal stream that powers downstream analytics. Wearable IoT biosensors such as smartwatches, ECG chest straps, PPG units, and EMG patches capture heart rate, heart-rate variability, blood-oxygen saturation, respiratory rate, and muscle activation during everyday activities [23,24]. On-device microcontrollers perform real-time denoising, motion-artifact rejection, and feature extraction, yielding compact metrics like RR intervals, SDNN, and RMSSD [25]. When PPG modules incorporate inertial measurement units, integrated motion cues further refine optical pulse estimates during exercise [26]. Structured entries from electronic health records supply longitudinal clinical context—diagnoses, laboratory panels, medication regimens, and clinician notes trace serial measures of blood pressure, HbA1c, and lipid profiles, guiding risk stratification and therapeutic decisions [27,28]. Molecular determinants enrich the phenotypic picture with genotype-informed insights: single-nucleotide polymorphisms, gene-expression signatures, and polygenic risk scores strengthen cardiovascular and metabolic models with personalized molecular factors [29,30]. Ambient sensors deployed in homes, workplaces, and public spaces continuously log environmental variables such as temperature, humidity, CO_2_, particulate matter, sound pressure, and illuminance, offering contextual covariates essential for equitable population-level monitoring [31,32]. Recent advances in optical sensing have significantly enriched the capabilities of the sensory layer, with photoplethysmography (PPG) emerging as a versatile modality for both contact and non-contact cardiovascular monitoring. Smartphone-based PPG systems, as demonstrated by Lovisotto et al., exploit subtle variations in reflected light to enable biometric authentication, though performance may degrade in cross-session scenarios due to physiological and environmental variability [33]. Extending beyond contact-based sensing, Yu et al. employed photoplethysmographic imaging (PPGI) to unobtrusively measure heart rate and heart rate variability in geriatric patients, achieving high accuracy in HR estimation (RMSE 0.48 bpm with RGB cameras, Sony Corporation, Minato, Tokyo, Japan) and moderate agreement in frequency-domain HRV indices [34]. Innovative sensor integration has also been realized in skin-like transparent sheets combining electroencephalography with camera-based PPG for simultaneous neurophysiological and hemodynamic assessment [35]. Consumer devices, such as smartphones, have proven capable of reliable HRV analysis: Zhang et al. validated rear-camera PPG against ECG, demonstrating strong correlations across most HRV metrics [36]. Beyond traditional vital sign monitoring, PPG signals can also encode motion-related information; Hnoohom et al. leveraged these artifacts within a deep residual architecture (PPG-NeXt) to enhance physical activity recognition [37]. Park et al. provided an integrative review of PPG signal generation mechanisms, measurement configurations, clinical applications, and noise-reduction strategies critical for robust sensory-layer design [38]. Advanced reconstruction further expands utility: Tang et al. presented a subject-based BiLSTM model that reconstructs long ECG waveforms directly from PPG without prior R-peak detection, reporting high correlation with reference ECG after alignment and successful long-segment synthesis from brief training windows [39]. Signal enhancement remains a key focus, as demonstrated by Botina-Monsalve et al., who applied LSTM-based deep filtering to remote PPG, and Kim et al., who combined support vector regression with deep learning to restore morphological fidelity to rPPG waveforms [40]. Collectively, these developments illustrate how PPG-based modalities can complement electrical, inertial, and environmental sensing in forming a rich, high-resolution input stream for cardiovascular digital twins. By harmonizing these heterogeneous inputs, the sensory layer delivers a coherent, high-resolution data stream that underpins advanced analytics and real-time decision-support systems.

Figure 2 schematically depicts how these heterogeneous streams merge at the edge: wearable, clinical, molecular, and environmental data undergo filtering, time-synchronization, and compression that reduces high-bandwidth raw signals to lightweight, time-aligned feature vectors.

A key challenge in aligning such heterogeneous cardiovascular data—such as photoplethysmography (PPG), electrocardiography (ECG), accelerometry, and contextual metadata from electronic health records (EHR)—is managing differences in sampling frequency and temporal resolution. Advanced synchronization strategies address this by first applying timestamp normalization to a common reference clock, often using the ECG R-wave peaks as temporal anchors for resampling lower-frequency signals such as PPG or respiration to physiologically relevant intervals (e.g., RR-interval alignment) [41]. Dynamic time warping (DTW) has been employed to handle subtle temporal drift, enabling flexible non-linear alignment between modalities in ambulatory settings where wearable clocks are unsynchronized [42]. Once aligned, feature-level fusion typically involves extracting modality-specific features (e.g., HRV metrics from ECG, pulse wave velocity from PPG, activity counts from accelerometers) followed by feature selection to remove redundancy and improve generalization. Cardiovascular studies frequently apply recursive feature elimination (RFE) with tree-based models such as XGBoost or Random Forests, minimum redundancy maximum relevance (mRMR) filtering [43], or mutual information–based ranking [44] to retain variables with the highest predictive contribution while avoiding collinearity between physiological and demographic inputs. For noise reduction, wavelet denoising [45] and empirical mode decomposition (EMD) [46] are widely used to suppress baseline wander, motion artifacts, and high-frequency noise in ECG and PPG without distorting fiducial points. In multimodal contexts, canonical correlation analysis (CCA) can also be used as a cross-modal filter—leveraging shared variance between two modalities to attenuate noise unique to one channel. In long-term monitoring, adaptive filtering with reference accelerometer channels effectively removes motion-induced artifacts from PPG [47].

The consolidated feed then migrates to cloud or on-premises platforms hosting AI models and patient-specific digital twins—virtual replicas that simulate disease dynamics and evaluate therapeutic scenarios in silico, thereby supporting personalized and preventive care pathways.

**Figure 3:**
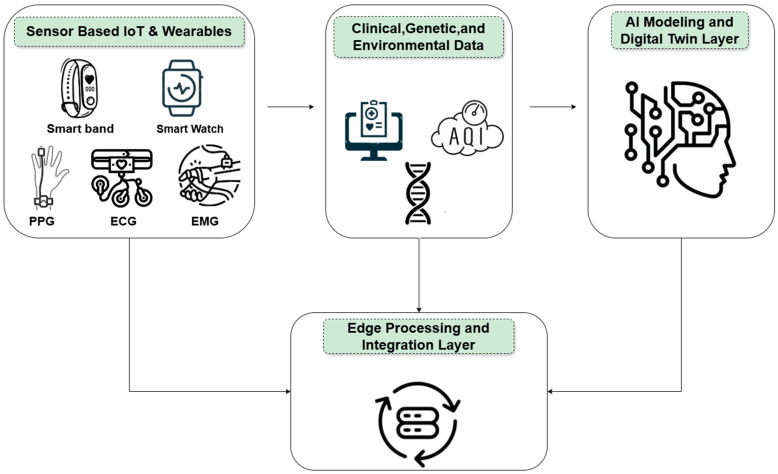
Multimodal sensor-to-AI pipeline: data from wearables and clinical–genetic–environmental sources are synchronized at the edge and forwarded to the AI/digital-twin layer, with feedback for continual recalibration.

Johnson & Saikia (2024) [10] provide a comprehensive synthesis of the wearable-to-digital-twin literature, outlining a three-stage pipeline comprising data acquisition, data processing, and model generation. Their review catalogs representative wearable sensors and highlights studies that have successfully implemented human–digital-twin workflows. The authors describe the central role of on-device microcontroller units in initial data handling, including collection, local buffering, and transmission. They emphasize the universal requirement for noise removal through analog and digital filtering as the first stage of processing, followed by the application of AI and machine learning techniques for pattern extraction and synthetic data generation in cases where direct measurements are limited. The review provides concrete examples of wearable sensors used in prior work, including ECG and EEG devices, inertial measurement units, EMG sensors, and textile-based or smart-clothing platforms. Notably, they reference specific studies that incorporated the MAX30102 optical module within integrated smart-clothing prototypes. The authors also draw attention to practical system constraints that should inform the design of the sensor–edge–cloud pipeline depicted in our Figure 3. These include limitations in computational resources, the necessity of continuous real-time acquisition to maintain up-to-date digital twin models, and the implementation of data security measures such as encryption during processing and transmission.

Table 1 complements the schematic by cataloging representative commercial and bespoke sensing platforms that implement this workflow. Although the devices differ in sampling frequency, modality breadth, wireless protocol, and on-board compute capacity, all conform to the acquisition-synchronization-transmission paradigm underpinning the sensory layer. Collectively, they provide the hardware backbone for continuous, location-agnostic monitoring, from high-resolution clinical-grade ECG recorders to low-power air-quality modules suitable for smart-home deployment.

Table 1 illustrates how heterogeneous sensor devices populate every tier of a multilayer health-monitoring architecture—from direct physiological signal capture on the body to cloud-based genetic analytics. First, the “Sensor Type” column spans four key modalities: electrical (ECG/EMG), optical (PPG/GSR/EDA), inertial (accelerometers), and digital (API streams, gas- and climate-sensors). Electrical channels (Zephyr BioHarness (Zephyr Technology, Annapolis, MD, USA), Polar H10 (Polar Electro, Kempele, Finland), Hexoskin (Carré Technologies Inc., Montreal, QC, Canada)) enable high-precision tracking of cardiac intervals and muscle activity; optical units (Empatica E4 (Empatica, Inc., Boston, MA, USA), Xiaomi Band 7 (Xiaomi Corporation, Beijing, China), Shimmer3 GSR+ (Shimmer Research, Dublin, Ireland)) provide lower-power yet continuous measurement of heart rate, SpO_2_ and galvanic skin response; inertial sensors enrich the picture with posture and activity data; digital and gas sensors (Winsen ZPHS01B (Winsen Electronics, Zhengzhou, China), Genomic API (Cloud Life Sciences API, Mountain View, CA, USA)) broaden observation to environmental exposure and genetic background, laying the foundation for truly personalized medicine. This diversity lets the system capture both intrinsic physiological fluctuations and extrinsic risk factors.

Second, “Measured Parameters” highlights disparities in data density. Professional devices (Zephyr (Zephyr Technology, Annapolis, MD, USA), Hexoskin (Carré Technologies Inc., Montreal, Quebec, Canada)) record RR-interval series and high-resolution respiratory signals—crucial for advanced HRV metrics such as RMSSD and SDNN. Budget fitness bands (Xiaomi Band 7, Xiaomi Corporation, Beijing, China) restrict themselves to instantaneous HR and step counts, but their mass adoption and high sampling frequency cover long-term population-level monitoring. Combining these sources supports simultaneous tracking of acute episodes (arrhythmias) and cumulative trends (sedentary lifestyle, chronic stress).

The third column, “Connectivity”, reveals a trade-off between power consumption and bandwidth. BLE and classic Bluetooth suit personal devices with hourly-to-daily recharge cycles, whereas Wi-Fi (Raspberry Pi (Raspberry Pi Ltd., Cambridge, UK) + MAX30102 (Analog Devices, Wilmington, USA)) provides continuous high-volume streaming in edge-server mode—appropriate for stationary scenarios or AI-prototype development. UART/I^2^C on the gas module underscores that environmental sensors are often embedded directly into smart-home IoT nodes and do not require a wireless link per sensor.

“Edge Capabilities” shows the shift from “raw” to “smart” devices. The simplest units (Winsen (Zhengzhou Winsen Electronics Technology Co., Ltd., Zhengzhou, China), Shimmer3 (Shimmer Research, Dublin, Ireland)) output unprocessed signals, relegating computation to higher layers. The next level—basic on-device filtering and buffering (Empatica (Empatica, Inc., Boston, MA, USA), Hexoskin (Carré Technologies Inc., Montreal, QC, Canada)). Finally, Zephyr, Polar, and a custom EMG node on ESP32 handle local RR-interval detection and muscle-fatigue classification, reducing latency and network load. This gradient of computational richness illustrates the evolution from “sensor as a cable” to “sensor as a mini-server”.

The “Role in Architecture” column combines these traits into semantic system layers. The Wearable Layer handles primary signal collection on the body. The Sensor + Edge Layer represents hybrids where part of the analysis (HRV, EMG classes) runs on-device. The Environmental Layer (CO_2_, temperature, humidity) adds living-context data essential for interpreting discomfort or sleep complaints. The Genetic Input Layer inserts constant individual predispositions (SNPs, PRS) into a unified timeline, closing the personalization loop from continuous bio signals to immutable genetic factors.

### 3.2. Machine Learning and Deep Learning for Data Interpretation and Prediction

Early research on computer-aided electrocardiography relied on statistical algorithms whose performance depended on hand-crafted features [48]. Growing computational power and the emergence of large open signal repositories have shifted the focus toward deep neural networks: a comprehensive survey of 1-D CNNs and recurrent hybrids applied to 12-lead ECGs documents, an exponential rise in publications, and a marked accuracy advantage over classical methods [49]. This trajectory culminated in the “AI-ECG’’ paradigm: a convolutional network from Mayo Clinic, trained on 50 000 ECG–echo pairs, detects left-ventricular systolic dysfunction with an AUC of ≈ 0.93 and even forecasts its onset in patients with normal imaging—an example of “disease foreseeing’’; a single-lead adaptation embedded in a digital stethoscope delivers a 15 s point-of-care screening [50].

Narrowing the target to critical conditions led to compact models: a five-layer CNN classifies normal beats, supraventricular, and ventricular ectopic beats with 98.33% overall accuracy [51], and a CNN-RNN cascade identifies acute myocardial infarction on 12-lead ECGs with 0.987 accuracy, surpassing expert cardiologists [52]. Further progress is tied to architecture that melds convolutions with attention: a transformer block inserted into a CNN lifts a multiclass F1 score to 0.786 by modeling long-range dependencies and employing link-constraint regularization to counter class imbalance [53].

Wearable sensors push monitoring beyond the clinic, streaming single-lead ECG, photoplethysmography, and accelerometry for multimodal analysis. A literature review (2018–2024) shows that deep-learning algorithms outperform traditional statistics in arrhythmia detection and heart-failure prognostication, with typical AUCs ranging from 0.85 to 0.95 [54]. In a textile prototype with an embedded electrode, a random forest detects coronary heart disease with 88% accuracy, highlighting feasibility in resource-limited settings [55]. An interpretable CNN-BiLSTM processing ECG, PPG and accelerometer data achieved an F1 of 0.92 for cardiovascular-risk stratification during real-world wear [56].

**Table 2:** Comparative assessment of AI methods for cardiac monitoring tasks.

Task (Cardiac Monitoring)	Method Class	Data (Brief)	Key Performance (Metric)	Where It Excels (Comparative)	Limitations/Caveats in CVD Use	Best-Fit Scenarios (Applicability)	References
LV systolic dysfunction screening (LVEF ≤ 40%) from 12-lead ECG	Transformer (foundation; pretrain → finetune)	Pretrained on 8.5 M ECGs; fine-tuned for LVEF/other tasks	AUROC 0.86 (internal with 1% labels); 0.87 external; boosts low-label tasks	Captures long-range temporal patterns; excellent label- efficiency; strong multi-task transfer	Requires massive pretraining compute; deployment still needs careful calibration/ interpretability	Population-scale ECG pre-screen → triage to echocardiography; low-label health-system settings	[57]
Near-term AF risk (≤14 days) from patch single-lead ECG	Deep learning (attention/temporal; multimodal)	459,889 ambulatory single-lead recordings (10 min–24 h)	AUC 0.80 (1-day horizon, “All features” model)	Early warning on AF-free ECG; integrates HRV + rhythm + demographics	Retrospective, device- specific; risk of distribution shift across wearables; prospective validation needed	Wearable AF surveillance and early-warning gating for patch/consumer ECG programs	[58]
Arrhythmia classification (12-lead; Chapman)	Graph Neural Network (lead-graph)	10,646 subjects; 12 leads; 7 classes	Accuracy 99.82%, Specificity 99.97% (GCN-WMI)	Encodes inter-lead relations; strong multi-lead performance	Limited for single-lead wearables; sensitive to lead configuration	In-clinic 12-lead analysis; multi-lead Holter/offline QA where inter-lead coupling matters	[59]
Coronary CTA: vessel extraction and anatomical labeling	GCN on vascular graphs	104 CCTA; 10 segment classes (AHA)	Tree-extraction 0.85; overall labeling 0.74	Preserves tree topology; better anatomical consistency than CNN-only	Dependent on reliable centerline/segmentation; calcifications/gaps still problematic	Pre-procedural planning; CAD quantification pipelines with human oversight	[60]
HF drug-response prediction/trajectory modeling (EHR)	Spatiotemporal GNN + Transformer (patient-visit graphs)	11,627 HF patients (Mayo Clinic EHR)	Outperformed baselines across 5 drug classes; best RMSE 0.0043 (NT-proBNP)	Learns longitudinal + relational patterns; subgrouping improves prediction and interpretability	Site-specific coding/ practice → transferability/ harmonization needed; privacy/PII governance	Hospital CDSS for HF titration; digital-twin personalization of therapy trajectories	[61]
Cardiac MRI segmentation (quality-aware automation)	Bayesian deep learning (uncertainty quantification)	Multi-center CMR; benchmarked Bayesian vs. non-Bayesian UQ	UQ triage cuts “poor” segmentations to 5%; only 31–48% cases require review	Safety guardrails; robust to OOD noise/blur (method- dependent)	Extra compute; needs workflow integration for human-in-the-loop review	Semi-automated CMR pipelines where safe triage and QC trump raw speed	[62]
Cuff-less blood pressure from wearables (PPG/ECG)	Transformer-hybrid (CNN+Transformer)	Two large wearables datasets: CAS-BP and Aurora-BP	CAS-BP: DBP 0.9 ± 6.5, SBP 0.7 ± 8.3 mmHg; Aurora-BP: DBP −0.4 ± 7.0, SBP −0.4 ± 8.6 mmHg; MAE below SOTA	Learns global temporal dependencies; fuses handcrafted + learned features	Domain/calibration drift across devices/skin tones/contexts; prospective ambulatory validation needed	Ambulatory BP trending and coaching with periodic calibration; patient-facing wearables	[63]

Table 2 summarizes the comparative evaluation of leading AI architectures for cardiovascular monitoring tasks, covering seven representative use cases from recent peer-reviewed studies [63]. Each row lists the target task, methodological class, key study, dataset scale, and headline performance metrics, followed by a critical assessment of where the approach excels and its primary limitations in cardiovascular deployment. The scenarios column outlines the most suitable application contexts for each method, linking technical capabilities to real-world use cases. Notably, transformer-based foundation models show exceptional label-efficiency and multi-task adaptability in large-scale ECG screening, while GNN architectures excel in capturing relational patterns in multi-lead ECG or vascular graph data. Bayesian deep learning methods provide safety guardrails through uncertainty quantification, making them valuable for semi-automated imaging workflows. Hybrid CNN–Transformer models for cuff-less blood pressure estimation demonstrate strong performance in wearable applications but face challenges related to domain drift and calibration across diverse devices and populations. This structured comparison directly addresses the need for a critical evaluation of AI methods in CVD applications, as emphasized by the review comments.

Personalization is now central to further gains. User-specific calibration of a stress-detection network elevates an LSTM classifier’s F1 from 60% to 91%, underscoring how physiological heterogeneity limits global models [64]. A two-stage “cloud-to-device’’ workflow, where a pre-trained CNN is progressively fine-tuned on local windows, improves activity recognition by ~8% while preserving energy efficiency on mobile hardware [65]. In distributed scenarios, personalized federated learning blends locally fine-tuned models with global experts, letting clients adapt to non-stationary streams without sharing raw telemetry [54]. A similar transfer-learning strategy, initializing a patient-specific logistic-regression model with global coefficients, boosts AUROC for acute kidney-injury prediction by 0.13 in high-risk subgroups, demonstrating the value of personalized initialization under sparse data [55].

Deep learning has thus transformed the ECG from a descriptive test into an active screening and prognostic tool; while fine-tuning and on-device learning lay the groundwork for truly personalized cardiology. Success in routine practice will hinge on balancing accuracy, interpretability, and scalability.

**Figure 4:**
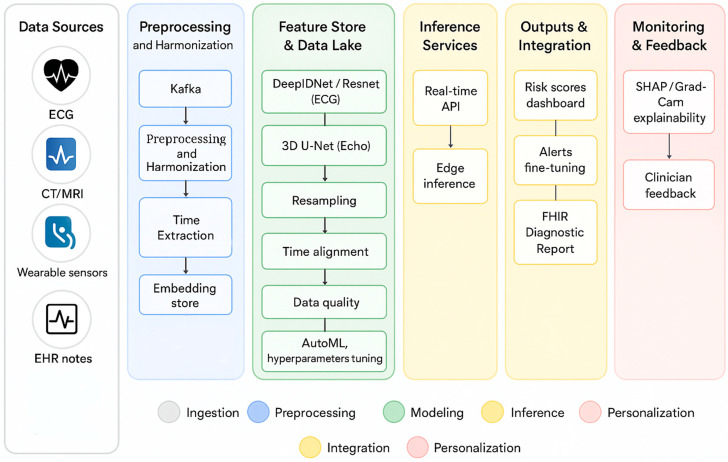
P-CVDNet: harmonized ECG/Echo/CT-MRI, wearable and EHR streams pass through feature stores and AutoML-tuned models to real-time/edge/batch inference, yielding risk dashboards, FHIR reports, alerts, and explainability with clinician feedback.

The pipeline illustrated in Figure 4 depicts a full-stack AI workflow for cardiovascular diagnostics and prediction. Multisource data from ECG, echocardiography, CT/MRI, wearable sensors, and EHR notes is ingested and harmonized using tools such as Kafka, DICOM Store, and FHIR Listener. Standardization, time extraction, and embedding storage prepare the data for modeling. Deep learning models—including DeepIDNet/ResNet for ECG and 3D U-Net for Echo—are supported by modules for resampling, alignment, and quality control. Feature processing leads to real-time and batch inference via APIs, with outputs integrated into clinical dashboards and FHIR-compatible reports. Personalization is enabled through explainable AI techniques (e.g., SHAP, Grad-CAM) and feedback loops, alongside federated and on-device fine-tuning that ensures patient-specific adaptation without transmitting raw data. An EchoNext (2025) study [66] represents a direct and large-scale implementation of the ECG-to–imaging-label supervised training strategy that underpins the P-CVDNet framework shown in Figure 3. In this approach, an ECG-based model is trained using echocardiographic measurements as ground-truth labels, enabling the generation of screening predictions that can be integrated into downstream clinical workflows. This methodology provides empirical support for the process depicted in Figure 3, specifically the pathway represented by the boxed arrow from “ECG input” through “Model training with imaging labels” to “Clinical dashboard/triage”. The study demonstrates how imaging-derived labels can enhance the diagnostic utility of ECG models, thereby facilitating more accurate risk stratification and decision-making in real-world care settings. A recent federated multimodal framework (2025) demonstrates integration of cardiac images, ECG signals, and clinical covariates via attention-based feature fusion, trains models using federated learning to preserve data locality, and implements node-level fine-tuning for local personalization—directly supporting P-CVDNet’s federated training and on-device/personalization components [67].

Extending beyond the ECG, unobtrusive PPG and smart-home signals provide the next layer of data-driven cardiovascular prediction. Machine learning (ML) and deep learning (DL) have become the cornerstone for interpreting complex multidimensional sensor streams and building prognostic models of cardiovascular risk. Modern continuous-monitoring architecture begins with contactless or minimally invasive data acquisition: wrist-worn PPG, seat-embedded ballistocardiogram sensors, and numerous smart-home devices generate flows linked to hemodynamics, rhythm variability, and behavior [68,69]. Signal variability caused by motion, illumination, and anatomy mandates comprehensive filtering; for PPG, this means adaptive motion-artifact suppression and illumination normalization [68].

During feature extraction, classical pulse-wave markers (PWV, PTT) are complemented by automatically learned descriptors. CNNs and transformers applied to raw PPG traces or spectrograms create deep latent representations that outperform hand-crafted features in estimating systolic and diastolic blood pressure [68]. For multi-sensor platforms (PPG + accelerometer + temperature) attention-based fusion has cut prediction MAE to 4–5 mm Hg on held-out cohorts [70].

With limited samples, three ensembles such as XGBoost remain robust thanks to mixed-feature handling and built-in importance metrics [71]. On large, densely annotated corpora, recurrent and transformer architectures able to capture long-range context dominate [70]. Behavioral predictors drawn from smart-home motion and appliance-current sensors increase hypertension-forecasting AUC by 4–6% [69].

Leave-one-subject-out validation is standard for generalizability [68,70]. In-home deployments, sliding personalized fine-tuning enables global models to adapt, reducing cuff-less BP error from 9–10 to 5–6 mm Hg after two weeks. Tree ensembles reveal pulse-wave-transit time and dicrotic-notch phase as top contributors [68], while SHAP/LRP analysis shows deep nets focusing on up-stroke slope and reflection-wave amplitudes—the physiological hallmarks of arterial stiffness [70].

Clinically, a wireless PPG cuff plus XGBoost flagged previously undiagnosed hypertension with 0.87 sensitivity and 0.82 specificity in a 150-participant home study [68], and smart-home activity trends predicted heart-failure decompensation 5–7 days before admission (AUC 0.78) [69]. The remaining challenges include ethnic and age generalizability and the need for periodic calibration [68]. Energy-efficient on-chip inference and privacy-preserving federated learning with differential noise already achieve comparable accuracy without transmitting raw bio signals [71]. Future directions point toward graph neural networks linking individual data to population risk maps and the creation of open longitudinal sensor datasets.

Personalization also accelerates through fine-tuning and retrieval-augmented large language models (LLMs). In the legal domain, adapting foundation models such as LLaMA to corpora of statutes, case law, and annotations enhances contextual sensitivity, curbs hallucinations, and enforces strict controls on personally identifiable information [72]. Supervised fine-tuning with a small but expertly annotated set can outperform larger synthetic corpora [73], while retrieval-augmented generation dynamically injects current regulations, cutting citation errors [74]. Techniques for quantifying memorization and selectively deleting confidential fragments without semantic loss are gaining adoption [75].

In personal data analytics, AI is shifting from population averages to individually profiled pathways. Hybrid recommender systems fused with deep NLP integrate genetic markers, disease history, and preferences to generate adaptive diet plans in real time [76]. Patient “digital twins’’ merge multimodal data and reinforcement-learning models to predict therapeutic response—for example, in orthodontic planning [77]. Personalized LLMs trained on precedents and client dossiers automate contract drafting and legal opinions, adjusting tone and argumentation to each customer’s history [78]. Dataset incompleteness and bias remain key challenges, driving validation on demographically diverse cohorts [79]. An emerging architectural trend is cascades of specialized models, orchestrated by a supervisory LLM that routes user requests to the optimal sub-module, thereby improving accuracy while reducing computational cost [80].

Table 3 presents a comparative overview of advanced fine-tuned deep learning models applied in cardiovascular diagnostics. The models leverage various input modalities, including ECG signals, ECG images, echocardiographic data, and real-time sensor information from IoT-based platforms. All models in this comparison are derived from or fine-tuned on domain-specific medical datasets and validated within peer-reviewed Q1/Q2 journals [81].

The architectures range from transformers and convolutional neural networks (CNNs) to ensemble and epistemic neural networks, each optimized for specific diagnostic targets such as arrhythmia, endocarditis, or broader cardiovascular disease (CVD) patterns. Notably, these models demonstrate high accuracy, ranging from 88.9% to 95.1%, emphasizing the clinical relevance of fine-tuning in enhancing diagnostic precision and model generalizability across heterogeneous patient populations.

Fine-tuning approaches include supervised learning with private ECG datasets, layer-specific re-training on clinical labels, and novel optimization strategies like the Boosted Sooty Tern algorithm. This reflects an ongoing trend in personalized medicine, where diagnostic algorithms are adapted to specific data characteristics and sensor configurations.

These models exemplify how deep learning can be precisely calibrated to meet clinical requirements, especially when integrated into wearable or IoT health monitoring systems.

## 4. AI Agents and Medical LLMs for Personalized Intervention

### 4.1. Generative AI and Medical LLMs

Generative artificial intelligence has rapidly reshaped biomedical research and clinical workflows, ushering in conversational agents such as ChatGPT 4.0 that can summarize literature, draft reports, and assist in decision-making [82]. Across scientific publishing, clinical documentation, and patient engagement, large language models (LLMs) now complement earlier machine-learning pipelines, expanding on foundations laid in pathology and medicine by classical predictive algorithms [83]. Their growing ubiquity in ophthalmology, radiology, infectious-disease surveillance, and mental-health support signals a broad shift toward data-driven, text-centric intelligence in healthcare [84,85,86].

**Figure 5:**
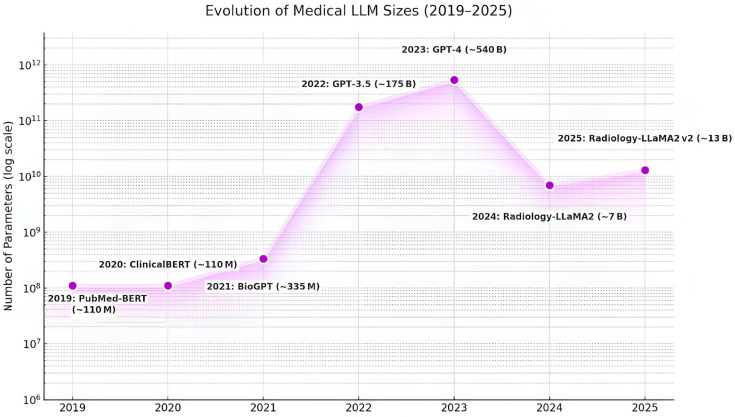
Medical LLM parameter counts by year, 2019–2025—sharp expansion through 2023 followed by a shift toward smaller, domain-adapted models for deployment.

Transformers form the computational backbone of modern LLMs; self-attention mechanisms introduced to overcome vanishing-gradient and long-dependency limits of RNNs and CNNs have enabled parameter scales exceeding hundreds of billions [87]. The graph presented in Figure 5 visually tracks this rapid expansion and subsequent optimization of parameter counts in key healthcare models, using a logarithmic vertical axis to compare models of vastly different scales. The timeline begins in 2019 with the PubMed-BERT model (around 110 million parameters) and the similarly sized ClinicalBERT in 2020 [84]. After a first notable upsizing with BioGPT (~335 M) in 2021 [88], a dramatic leap occurred, marking the beginning of a “size race”: in 2022, GPT-3.5 reached 175 billion parameters [87], and in 2023, this trend reached its apex with GPT-4, estimated at a massive 540 billion parameters [82]. However, a fundamental shift is observed after this peak: from 2024 onward, specialized “lightweight” architectures gain prominence, such as Radiology-LLaMA2 (~7B parameters) and its enhanced v2 version (~13B) by 2025 [89]. This pivot signifies a paradigm shift in AI development for medicine—the industry is moving away from creating enormous, general-purpose models and is instead focusing on more efficient, less costly, and highly specialized solutions for specific domains, such as radiology.

Pre-training on vast mixed-domain corpora such as web content, PubMed and de-identified electronic health records establishes powerful priors that are later adapted to clinical language through domain-specific fine-tuning [90,91]. Parameter-efficient tuning strategies—including Low-Rank Adaptation (LoRA) combined with DeepSpeed tensor parallelism—lower hardware barriers for hospitals and SMEs while preserving quality [92]. Retrieval-Augmented Generation, rule-guided prompting and hybrid encoder–decoder schemes mitigate hallucinations and inject domain knowledge, improving factual reliability in diagnostic dialogs [93,94,95].

Integrating Medical LLMs into a Digital Cardiovascular Twin Framework Recent work on generative AI for personalized medicine shows that domain-adapted LLMs can fuse heterogeneous data streams—including textual reports, sensor feeds and imaging metadata—into coherent patient-specific knowledge graphs that drive downstream simulations [88,96]. Within a digital cardiovascular twin, these models act as the semantic layer that continuously ingests new clinical notes, echocardiography findings and wearable HRV summaries, converting them into structured inputs for multiscale hemodynamic solvers. Parameter-efficient fine-tuning (e.g., LoRA + DeepSpeed) lowers the hardware burden for hospital servers, enabling real-time updates of twin parameters at the bedside [92]. Retrieval-augmented generation and rule-guided prompting further curtail hallucinations, ensuring that automatically generated boundary conditions remain physiologically plausible before propagating through the twin’s differential-equation core [95,96]. Privacy-preserving techniques—federated learning, differential privacy and homomorphic encryption—guard sensitive cardiovascular data while still permitting federated calibration of population priors that make the twin robust across demographics [97]. In this way, the same transformer backbone that underpins large-scale clinical summarization (Figure 3) also becomes the connective tissue between raw multimodal evidence and dynamical patient-specific modeling, moving digital twins from static anatomical replicas to continuously learning, decision-support companions.

Generative architectures beyond autoregressive decoders reinforce the ecosystem: generative adversarial networks and diffusion models synthesize realistic medical images and privacy-preserving patient records for data augmentation and personalized-medicine pipelines [87,96]. Variational and denoising auto-encoders underpin anomaly detection and latent representation learning in imaging and signal analysis [97]. Robust deployment further demands differential privacy, federated learning and homomorphic encryption to guard sensitive data, while emerging open-weight models such as Yi 34B facilitate reproducible benchmarking and workflow integration [98,99]. Cross-lingual pre-training broadens accessibility; comparative studies show GPT-4 outperforms earlier versions when reasoning over non-English clinical questions, highlighting the value of multilingual grounding [85].

Domain-adapted LLMs already demonstrate tangible impact. In surgical pathology, foundation models accelerate report generation and triage, integrating seamlessly with microscope-based whole-slide imaging pipelines [85]. This Table 4 synthesizes ten representative entries by application domain—from clinical communication and summarization [82] to pathology diagnostics [83], ophthalmology workflows [84], broad generative-AI surveys [87], multimodal healthcare integration [88], medical image classification and EEG analytics [94], multilingual LoRA-enhanced chatbots [100], visual question-answering systems [90,100], clinical text summarization [90], and mental-health chatbot evaluation [101,102]—alongside their core architectures (decoder-only transformers [82], BERT-derived encoders [84], GANs and diffusion networks [87], LoRA + DeepSpeed adaptations [92], and encoder–decoder hybrids [90] and key functional features, with each row linked to its source referent.

Ophthalmology researchers employ medical-tuned GPT-variants to draft referral letters and analyze retinal findings, while simultaneously benchmarking diagnostic aptitude on board-style examinations [84]. Visual-question-answering frameworks couple radiographic inputs with textual reasoning to produce explanatory responses for clinicians and patients like [100].

Oncology and radiology services have begun to couple domain-adapted LLMs with ambient “digital–scribe” technology to auto-summarize radiotherapy protocols and draft personalized treatment plans, thereby accelerating multidisciplinary decision-making processes [86,89,103]. A controlled study in a simulated vitreoretinal clinic demonstrated that ChatGPT-3.5 transcribed encounter audio with 96.5% accuracy and generated notes that attained 87% of the maximum Physician Documentation Quality Index (PDQI-9) score, significantly outperforming Google Gemini 1.0 Pro, yet still exhibiting occasional hallucinations that necessitate clinician oversight [86]. Beyond the consultation room, AI-driven message-triage systems and auto-drafted patient replies have shortened first-response times by approximately one hour and offer a pragmatic route to reducing the electronic-health-record (EHR) burden that contributes to clinician burnout [104].

Expert-panel discourse stresses that such efficiency gains must be balanced against requirements for transparency, reproducible validation, and the establishment of shared national testbeds to guarantee reliable medical AI [105]. Delaying deployment also imposes a measurable opportunity cost: postponing intensive-care-unit AI tools forfeits predicted improvements in sepsis prediction, administrative efficiency, and equitable access to care [106]. Safety-net providers similarly view multilingual chatbots, automated documentation, and 24/7 AI-based triage as near-term levers for expanding access among underserved populations, provided that algorithmic bias is continuously audited and mitigated [107].

To align innovation with governance, a four-pillar vendor-assessment framework—strategic alignment, executive sponsorship, value/impact analysis, and a 12-factor risk assessment—has been proposed; generative systems are classified as highest-risk owing to their propensity for hallucination and the potential clinical harm that may ensue [108]. Commercial deployments already reflect these considerations. Nabla Copilot, an ambient AI assistant used by ≈85,000 clinicians across more than 130 healthcare organizations and integrated with ≥20 commercial EHR platforms, exemplifies the transition of LLM scribes from pilot studies to routine practice [109]. Likewise, the Oracle Health Clinical AI Agent reports an average 30% reduction in daily documentation time while supporting more than 30 specialty areas, confirming the scalability of generative documentation within production EHR environments [110]. Taken together with synthetic-patient generators that create privacy-preserving, balanced cohorts for precision-medicine discovery [96], these developments indicate that transformer-based generative models—fortified by robust risk-management frameworks and domain-specific adaptation—are advancing from experimental prototypes toward dependable collaborators throughout the continuum of healthcare delivery.

Concrete clinical deployments demonstrate how language-centric and language-linked AI can operate within a cardiovascular digital-twin loop. EchoNext, a deep-learning system trained on large-scale pairs of routine 12-lead ECGs and echocardiography outcomes, identifies structural heart disease directly from ECG and enables a pragmatic “sensor → AI triage → echocardiography” workflow; although not an LLM, it supplies the sensing/inference backbone onto which twin-layered reasoning and reporting can be built [66,111,112]. Medical LLMs for echocardiography reporting automatically draft guideline-aligned summaries, translating model states and imaging findings into clinician-ready documentation and patient-facing explanations [113]. In parallel, a wearable ECG–echo foundation model transfers echocardiography semantics into single-lead encoders for at-home monitoring, bridging continuous sensing with echo-level phenotypes and supporting longitudinal risk tracking and label-efficient learning within the twin [81,84]. Together, these components instantiate a practical pathway from ubiquitous sensing through AI-driven triage and cross-modal inference to LLM-mediated reporting and communication, addressing the current gap in real-world applications.

### 4.2. Proactive AI Agents

The development of AI agents has progressed steadily over several decades, driven by theoretical breakthroughs and the practical need for autonomous intelligent systems that can operate in complex real-world environments. Researchers have long understood that building effective AI agents requires integrating multiple disciplines, including formal logic, cognitive science, machine learning, and software engineering—so that these agents can not only make autonomous decisions but also behave reliably and explainably [114]. The primary motivation for creating AI agents is to replicate aspects of human cognition—such as perception, reasoning, planning, and learning—in a computational setting while ensuring scalability and interoperability across diverse application domains [115]. This report surveys the core technologies for building AI agents, encompassing logic-oriented methods, agent-oriented programming languages, cognitive architectures, emerging paradigms based on large language models (LLMs), multi-agent communication frameworks, simulation environments, and integrated intelligent platforms [116].

Logic-based AI methodologies constitute one of the earliest and most enduring paradigms for specifying intelligent agent behavior. These approaches employ formal logics—propositional, first-order, modal, temporal, executable, and deontic—to encode an agent’s knowledge base, decision-making constraints, and reasoning processes. Through logic programming, developers can formally verify agent-behavior properties, ensuring a high degree of explainability and traceability—capabilities that are especially critical in safety-sensitive applications. Yet, despite the theoretical elegance and expressiveness offered by logical systems, systematic reviews indicate that only a subset of these technologies has been modernized recently, highlighting challenges in industrial deployment and maintenance. Logic-based agents typically rely on frameworks that implement the Belief–Desire–Intention (BDI) model, providing structured means to represent mental states and govern agent behavior through logical inference rules [114].

Cognitive architectures are frameworks designed to model and simulate human cognitive processes within AI agents, effectively bridging the gap between abstract reasoning and embodied behavior. Well-known architectures such as SOAR, ACT-R, and CLARION offer comprehensive models of cognition, integrating modules for perception, memory, decision-making and learning. These architectures decompose cognition into interrelated components: working memory processes transient information, long-term memory stores acquired knowledge, and a central executive system coordinates cognitive operations. The practical value of cognitive architecture lies in its ability to produce agents that display adaptive, context-dependent behavior, making them suitable for applications in robotics, interactive tutoring systems and personal assistants. Cognitive models not only address how cognitive processing occurs but also provide experience-based learning mechanisms, allowing agents to refine strategies through feedback from the environment [117].

Remote health-monitoring systems provide continuous real-time collection of patients’ vital signs [118]. Wearable and wireless sensors capture heart rate, blood pressure, body temperature, respiratory rate and blood-oxygen saturation, while smartwatches and fitness trackers additionally track activity level, sleep, calorie expenditure and stress [118,119]. The IoT and IoMT infrastructure merges data from multiple sources, including wireless body-area networks, enhancing diagnostic and prognostic accuracy. Extended monitoring covers environmental parameters, gait analysis for Parkinson’s disease, and geolocation of potentially infected patients [120,121]. Integrated platforms such as the Integrated Intelligent Long-Term Care Service Management System consolidate all data in a single interface, giving a comprehensive overview of older patients’ status [122].

Intelligent alerts accelerate medical intervention [121]. Systems automatically transmit data to physicians in real time and flag falls or deviations in vital signs [121,122]. Chatbots powered by large language models engage patients in dialog, answer questions, and provide medication advice, while integration with electronic health records ensures instant notifications for clinicians [123]. Using the collected data, the systems generate personalized lifestyle recommendations [120,122]. AI models enriched with expert databases and nutritional information create individualized dietary plans that consider age, sex, weight, and medical history [121,122].

Advanced digital healthcare solutions draw on integrated continuous-monitoring data to propose lifestyle adjustments that can prevent further deterioration of chronic diseases. In diabetes care, the systems analyze how diet and physical activity influence glycemic trends, then suggest individualized measures that may include specific exercise routines, dietary changes, or insulin dosage adjustments [103,104,105,106,107,108,109,110,111,112,113,114,115,116,117,118,119,120,121,122,123,124]. These suggestions are produced by algorithms that combine longitudinal patient data, patient-reported outcomes, and established clinical guidelines, fostering a more proactive approach to disease management [125,126]. Recommendations are seamlessly embedded in digital health-app interfaces, where users view visual dashboards of their health trends with overlaid actionable advice [126]. This integration not only underscores the importance of ongoing self-care but also empowers patients through education and behavioral incentives [127].

In healthcare, artificial intelligence (AI) already has numerous real-world applications, including AI-agent concepts such as those developed by Oracle and Ambient AI, transforming medical services and health-system management [128]. Oracle is actively advancing AI in healthcare along several key lines. First, the company is building a digital-twin platform for therapeutic processes: virtual replicas of real-world objects, systems or procedures are constructed from real-time data, enabling continuous monitoring, analysis, and modeling of treatment to optimize patient care and boost operational efficiency [129]. Second, Oracle is investing in generative AI for electronic health records, aiming to raise care quality, expand data accessibility, streamline clinical workflows, and reduce physicians’ administrative burden; relevant use cases include data management, patient engagement, clinical decision support, and predictive analytics [130]. Third, in partnership with IBM and Accenture, Oracle is deploying AI systems for personalized professional training that deliver on-demand learning and real-time coaching, extending the capabilities of virtual mentors [131].

An AI agent is a software entity capable of perceiving and interpreting its environment, making decisions based on incoming information and acting—autonomously or semi-autonomously—to achieve specific goals [132]. A typical example in healthcare is a virtual health assistant that checks symptoms, schedules appointments, issues medication reminders and provides mental-health support [133].

Ambient Intelligence (AMI) in healthcare represents a paradigm of intelligent, context-aware environments that adapt to each patient’s unique needs. Integrating AI with the Internet of Medical Things (IoMT) amplifies AMI by allowing systems to sense, process and respond to patient data without explicit commands, thereby realizing the concept of “smart living” [134]. In AMI environments, sensors, actuators and AI algorithms jointly work to continuously assess the user’s condition and unobtrusively adjust surroundings when necessary [135]. Ambient Intelligence Assisted-Living settings include hospital wards, where AMI monitors ICU patients’ vital signs, forecasts length of stay to prevent ED overcrowding and fine-tunes lighting or temperature for comfort [134]; clinics and ambulatory facilities, where IoT sensors and AI analytics optimize patient flow, cut waiting times and personalize treatment pathways [136]; and smart homes, where AMI systems support daily activities, track health status and deliver tailored care for older adults, people with disabilities and children—capabilities that proved especially valuable during the pandemic [137].

Proactive AI agents operate by autonomously collecting, analyzing, and reacting to real-time data streams from diverse sources. These agents combine predictive models, sensor networks, and large language models to provide continuous clinical support, enabling early disease detection, personalized risk assessment, and timely therapeutic interventions. Moving beyond discrete diagnostic tasks, they integrate seamlessly into clinical workflows and operational systems to optimize patient management. AI agents continually gather biometric data via wearable sensors, process EHR information, and automatically alert physicians when early signs of health deterioration are detected [138,139]. Advanced techniques—including digital-twin models—facilitate patient-specific disease-trajectory modeling, thereby enhancing the personalization of treatment strategies in cardiovascular medicine [140].

Integrating conversational agents into digital healthcare platforms delivers continuous support for managing chronic diseases, including cardiovascular conditions. These agents leverage natural language processing and large language models to engage patients in self-monitoring, provide personalized recommendations, and ensure adherence to treatment protocols. Although current adoption is concentrated in other chronic illnesses such as type II diabetes, the same technologies are poised to extend into cardiology, supporting patients with heart failure, hypertension, and arrhythmia management [141]. Within this context, proactive AI agents will not only assist in diagnosis but also strengthen patient engagement and treatment adherence, both of which critically influence long-term cardiovascular outcomes.

The advent of digital twin models in cardiology has paved the way for a new era of personalized medicine. By simulating patient-specific cardiac physiology using comprehensive datasets, these models facilitate predictive analytics that can forecast disease progression, evaluate responses to therapy, and aid clinicians in tailoring interventions with unprecedented precision. These digital twin models, when integrated with AI-powered diagnostic tools, can offer a dynamic, continuously updated representation of a patient’s cardiac health, thereby optimizing treatment pathways and minimizing invasive procedures [140].

## 5. Discussion

Recent cardiology overviews consolidate layered cardiovascular digital twins that integrate multimodal sensing, hybrid mechanistic–AI modeling, and closed feedback loops [11]. In cardiac electrophysiology, state-of-the-art reviews in cardiac electrophysiology describe continuous data integration, patient-specific calibration, and decision support within digital-twin workflows [142]. Foundational precision-cardiology work argues for combining mechanistic simulators with data-driven models as the basis for patient-specific twins [143]. Population-scale cardiac twins have been generated from UK Biobank imaging and ECG, demonstrating feasibility at the cohort scale [144]. Virtual pacing on patient-specific twins has predicted response to cardiac resynchronization therapy [20].

**Figure 6:**
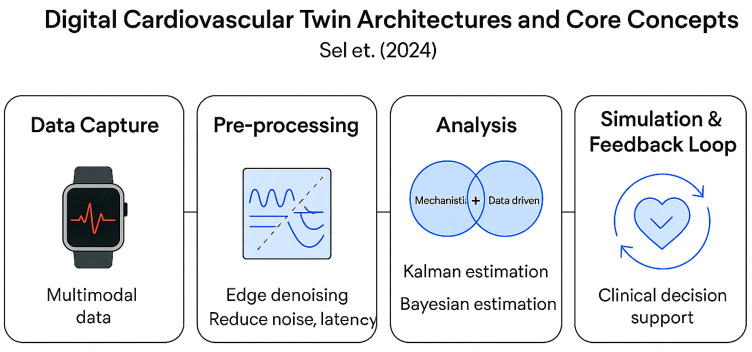
Architecture of a cardiovascular digital twin by Set et al. [11].

**Figure 7:**
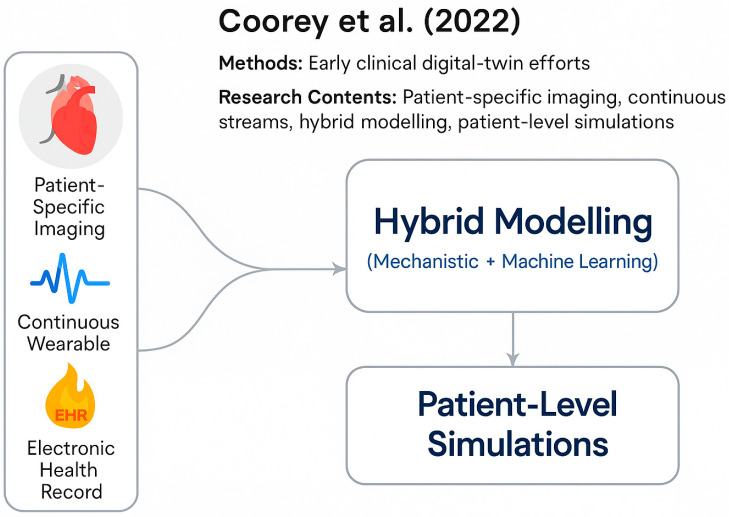
Coorey et al. (2022) framework showing integration of multimodal patient data for cardiovascular simulation [8].

Current literature provides clear and converging roadmaps for developing clinically impactful digital twins. Sel et al. (2024) [11] present an engineering roadmap for cardiovascular digital twins that closely aligns with the four-layer architecture shown in Figure 6. Coorey et al. (2022) [8], as shown in Figure 7, review early clinical digital-twin efforts and argue that patient-specific imaging plus continuous wearable and EHR streams are necessary to build clinically meaningful twins.

**Figure 8:**
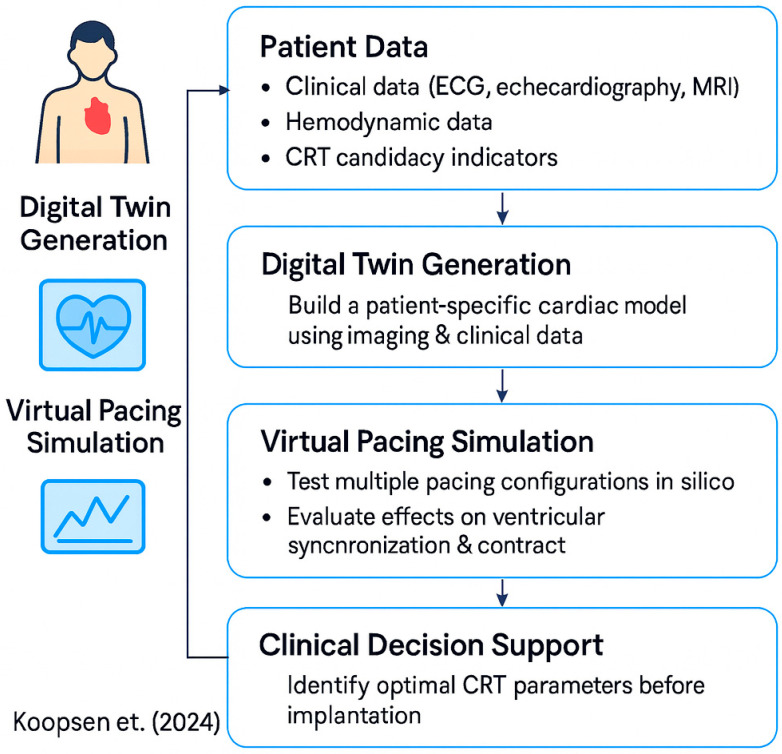
Koopsen et al. (2024) digital twin workflow for CRT optimization [20].

As a clinical example, Koopsen et al. (2024) [20] use a patient-specific digital twin to optimize CRT. Using ECG, echocardiography, MRI, and hemodynamic measurements, they construct a digital heart twin, run virtual pacing simulations, and select optimal CRT settings prior to implantation, demonstrating the practical application of the simulation layer in personalized cardiovascular interventions (Figure 8).

A persistent challenge in advancing digital health systems is ensuring the accuracy, reliability, and interoperability of wearable devices and Electronic Health Records (EHRs) across diverse clinical settings and patient populations [145]. Although wearables enable continuous monitoring and personalized intervention, unresolved interoperability issues limit their effectiveness. Enhancing interoperability and establishing standardized communication protocols remain critical to mitigating data silos and enabling seamless healthcare delivery through integration with EHRs [146]. However, EHR management and exchange are hindered by fragmented systems, inconsistent standards, and security concerns, often delaying treatment due to a lack of timely data. Distributed Ledger Technology (DLT) has been proposed to enhance security, integrity, and transparency, but its widespread adoption is constrained by scalability issues, early-stage development, and the absence of standardized implementation methods. Additionally, cross-platform interoperability among blockchain systems remains unresolved [147]. Standards like HL7 FHIR are valuable but insufficient for patient-centered interoperability, especially when records are distributed across providers with differing implementations. This limitation is particularly critical in emergency and cross-border contexts. Barriers such as inconsistent standard implementation, governance challenges, and the high cost of customizing data pipelines further complicate EHR integration [148]. Efforts in domains like cardiology have used HL7 CDA, v2, and FHIR to integrate systems within hospital infrastructures and regional health networks, enabling communication across subsystems such as PACS, RIS, and booking platforms. Nonetheless, administrative resistance to blockchain adoption—due to concerns over distributed data control—remains a challenge [149].

In the context of IoT medical devices, interoperability is complicated by heterogeneous data formats. A promising solution involves evaluating data quality and transforming only high-quality input into HL7 FHIR-compliant formats using structural and semantic mapping. Yet, broader challenges in creating unified frameworks for both data quality and interoperability persist [150]. Existing frameworks also fall short in addressing technical, semantic, and regulatory concerns. Inconsistent terminology undermines semantic interoperability, while blockchain-based approaches face limitations such as storage costs, scalability issues, and inadequate support for privacy rights like the GDPR’s “right of erasure.” The difficulty of adapting these frameworks to varied institutional contexts limits their broader adoption [151]. Cloud-based platforms demonstrate the potential of interoperability and automation to enhance clinical efficiency. However, current EHR implementations still face design inconsistencies, limited inter-system communication, and a lack of clinical decision support, even when using FHIR and SMART on FHIR standards [152]. Heterogeneous wearable ecosystems face interoperability challenges due to fragmented markets and varying data models, sleep terminology, and counter reset practices across platforms like Apple Health, Google Fit, Fitbit, and others. To overcome these issues, the proposed Analytic Engine includes a Data Homogenizer that standardizes data structures, assigns consistent temporal labels, manages resets, and handles missing data via imputation or smartphone fusion. A four-layer framework—connectivity, data, authentication, and user—further supports scalable, cross-platform integration of wearable health data [153]. Another research proposed a metamodeling-based approach to improve IoT interoperability by formally modeling device behaviors, protocols, and interactions. It creates a unified framework that supports the integration of heterogeneous components. Automated tools like Acceleron generate test cases from these models to validate interoperability efficiently. The approach enhances system reliability, reduces testing effort, and ensures consistent communication across diverse IoT systems [154].

Decentralized Clinical Trials (DCTs) increasingly utilize Digital Health Technologies (DHTs) to improve patient safety through real-time monitoring and prompt detection of adverse events, especially in vulnerable populations. However, risks remain due to inaccurate measurements and the need for in-person procedures like cardiac auscultation. Regulatory guidance for DCTs is still evolving, requiring close collaboration with authorities such as the FDA. Global data privacy variations—such as HIPAA in the United States and GDPR in the European Union—complicate multi-regional trials, and automated data flows can create issues if patients contact sponsors before clinicians, due to anonymization rules [155]. Training for participants and local healthcare professionals enhances remote AE reporting, though challenges like device failures, cognitive limitations, and poor connectivity persist. Agencies have issued broad guidelines on DCT design and DHT use, but Statistical Analysis Plans (SAPs) must account for remote data limitations and potential malfunctions. Alignment with the FDA and EMA on design, safety, and privacy protocols is critical for regulatory approval [156]. DCT suitability depends on the Investigational Medicinal Product’s (IMP) risk profile, with well-characterized drugs preferred over new molecular entities. The Clinical Trials Transformation Initiative (CTTI) identifies additional barriers, including inconsistent state-level telemedicine laws, cross-state healthcare provider licensing, and complex IMP shipment and accountability processes [157]. In trials evaluating Digital Therapeutics (DTx), safety is maintained through exclusion of high-risk participants and physician-classified AE reporting. Electronic tools for identifying device malfunctions also enhance safety. Still, regulatory guidance for DTx and Software as a Medical Device (SaMD) remains vague, with overlapping requirements and regional inconsistencies complicating standardization [158]. Both the FDA and EMA emphasize that decentralized trial elements must be assessed based on patient safety, data integrity, trial type, drug profile, and study complexity. Most DCTs reviewed involve low-risk, self-administered interventions for chronic or preventive conditions, consistent with these guidelines. However, inconsistent terminology in clinical trial registries—using terms like “remote,” “virtual,” and “hybrid” instead of “decentralized”—complicates identification and analysis. Concerns also remain about the adequacy of technological infrastructure and personnel training to maintain safety and data quality [159].

The integration of AI into healthcare raises key concerns around ethics, privacy, and trust. Core principles—beneficence, autonomy, justice, transparency, and accountability—underpin regulations like the EU’s Medical Device Regulation (MDR) and AI Act, which govern high-risk systems. Challenges include reduced doctor–patient trust, clinician deskilling, and unclear accountability for AI-driven errors [160]. Despite its benefits, risks such as data leakage, algorithmic bias, and fabricated output (hallucinations) present serious limitations, particularly in clinical decision-making. The success of AI systems depends not only on technical soundness but also on public trust, which varies based on demographic factors such as age, education, and prior technological exposure Adequate training—ranging from undergraduate education to professional development—is critical to ensuring appropriate use and reducing risks related to liability, misinterpretation, or improper delegation of clinical authority [161].

Ethical challenges in AI can be grouped as epistemic (uncertain, opaque, or inaccurate outputs), normative (fairness and harm prevention), and overarching (transparency and accountability). A key concern is the opacity of AI in high-stakes care, which hinders accountability and fosters automation bias. Over-reliance on AI may erode clinical expertise and widen healthcare disparities [162]. Legal reviews show that regulations like GDPR face limitations, especially with data ownership, genetic data, and inequality in low-resource settings. Ethical goals like diversity and fairness are hard to achieve with uneven data. Practical barriers—such as data cleaning, ethical approvals, and perceived intrusiveness—can hinder fair and effective AI deployment [163]. Legal and social issues in AI healthcare adoption should include accountability for errors, informed consent, and preventing discrimination. AI should assist—not replace—clinicians. Equitable systems require diverse data, ongoing validation, strong cybersecurity, and public trust through transparency [164]. To address AI’s ethical challenges, experts advocate for actionable strategies with measurable metrics like explainability scores and bias reduction. A trustworthy AI ecosystem should prioritize transparency, fairness, safety, and autonomy. Tools such as SHAP and LIME improve interpretability, while adaptive regulations are needed to keep pace with AI’s evolution. Current frameworks lag in handling real-time updates and sustainability, prompting calls for “ethical by design” development from the outset [165].

Further complexity arises from the need to protect sensitive health data across the entire AI lifecycle. Unauthorized access, re-identification, and misuse of personal data remain significant threats. Privacy-preserving technologies such as Homomorphic Encryption, Secure Multiparty Computation, and Trusted Execution Environments are being explored to mitigate these risks. However, balancing strong security with model interpretability remains a challenge. Explainable AI is essential to build trust among clinicians and patients. A shift from data ownership to data stewardship is encouraged to support ethical practices. Bias in training data and model outputs must be urgently addressed to prevent misinformation and inequality [166]. Such ethical concerns intersect with the technical limitations of LLMs, which suffer from hallucinations, inconsistent performance, hallucinations, inconsistent performance, and poor integration with legacy systems. Evaluation is hindered by a lack of clinical benchmarks and validation protocols. Their opacity raises ethical concerns around bias, accountability, and informed consent. While powerful, LLMs should support—not replace—clinical judgment, with clinicians maintaining final responsibility [167]. In practice, especially in emergency settings, AI shows promise for faster diagnostics and personalized treatment. However, its success depends on diverse training data, clinician collaboration, and careful integration to avoid dehumanizing care and diminishing empathy [168]. Technically, tuning models and capturing clinical reasoning is complex. Humanistic concerns like autonomy and empathy are hard to replicate, and AI’s opacity can undermine shared decisions. Legal uncertainty around liability further impedes adoption. Building trustworthy AI requires training developers, validators, and clinicians to ensure ethical, safe, and effective use [169].

As demonstrated in this review, medical LLMs are already being successfully deployed in real-world clinical settings to optimize specific workflows. Commercial solutions like Nabla Copilot and the Oracle Health Clinical AI Agent have proven effective in reducing the administrative burden on physicians by automating clinical documentation, thereby improving efficiency. These applications represent a significant step forward in applying AI in healthcare. However, a critical distinction must be made between these standalone, task-specific applications and the full integration of LLMs into a comprehensive cardiovascular digital twin framework. While the former are already in clinical use, the latter remains largely in the conceptual and simulation phase.

Cardiovascular digital-twin frameworks aim to construct patient-specific virtual replicas of cardiac structure and function by integrating heterogeneous data streams, including three-dimensional cardiac MRI, electrophysiological measurements, and even multi-omics profiles [170]. Their capacity to simulate electrocardiographic patterns and forecast treatment responses represents a paradigm shift for precision cardiology. Nevertheless, despite the maturity of multiple modeling approaches, translating these twins from research prototypes into routinely deployed clinical decision-support systems remains challenging [171,172].

A substantial body of reports indicates that although general-purpose large language models (LLMs)—even when further adapted to medical corpora—perform strongly on standardized benchmarks, a persistent gap remains between benchmark performance and real-world clinical validation [171,172,173]. Specifically for the integration of such LLMs into cardiovascular digital-twin frameworks, studies repeatedly note that results are derived largely from simulated environments or small-scale experimental datasets rather than from genuine clinical deployments [170]. Numerous investigations present modeling results that highlight the prospective benefits of using medical LLMs within digital-twin settings. For example, TWIN-GPT has demonstrated high-fidelity simulation performance when reproducing clinical-trial data and outcomes for virtual patients [174]. While promising, TWIN-GPT is explicitly positioned as a tool for synthetic-data generation and for supporting virtual clinical trials, not as a system implemented in routine care. Indeed, the study states that, although the model improves outcome prediction, it does not provide concrete evidence of integration into operational cardiovascular digital-twin frameworks [175].

Similarly, work exploring LLM-based generation of clinical reports or patient-facing communication shows that model outputs can approach expert-level quality under controlled conditions, yet full integration of such systems into cardiovascular digital-twin workflows remains unproven [173,175]. Notably, investigators emphasize the need for extensive clinical validation—including randomized controlled trials and testing under real-world conditions—before these applications can be regarded as reliable [172,176].

A recurring theme across recent publications is the explicit acknowledgement that, to date, no study has reported successful augmentation of cardiovascular digital-twin frameworks with medical LLMs in real clinical settings. One report states unequivocally that “there is no evidence that LLMs, when used within a cardiovascular twin framework, have yet been integrated or validated on real-world clinical cases” [172]. This view is echoed by several other works that point to the divergence between benchmark scores and clinical deployment [172].

Synthesizing the surveyed literature yields consistent observations across independent studies. Several works [172,173,174] explicitly note that while cardiovascular digital-twin frameworks have been widely examined from a modeling standpoint, the incorporation of LLMs into these systems has not yet been demonstrated on real clinical use cases. Complementary studies focused on technical aspects of medical LLMs [172] concur that integration remains experimental. Even reviews dedicated to multimodal integration and digital-twin methodology [170] clearly remark on the absence of real-world clinical augmentation by LLMs in cardiovascular applications. Finally, investigations discussing continued pretraining and domain adaptation [176] reaffirm that, despite model advances, the lack of external clinical validation is a persistent theme. Taken together, these findings support the conclusion that, although foundational research is encouraging, no study has yet substantiated the clinical augmentation of cardiovascular digital-twin frameworks with medical LLMs [176].

While EchoNext demonstrates clinical-grade ECG triage for SHD [66] and is being advanced toward real-world deployment [111], it is a deep-learning classifier rather than an LLM. We therefore pair it with medical LLMs for documentation and communication [115], and with cross-modal foundation encoders to extend reach to single-lead wearables [81]. Future work should test these components prospectively within a single twin stack, measuring not only AUC but workflow impact (time-to-echo, detection yield) and safety.

## 6. Conclusions

This narrative review synthesized 183 peer-reviewed publications from 2016 to 2025 retrieved via PubMed, MDPI, Scopus, IEEE Xplore and Web of Science, charting the stepwise evolution of the digital cardiovascular twin. Section 1 draws on 22 papers that outline the limitations of reactive cardiology, the emergence of a proactive paradigm and the foundational concept of a continuously updated virtual heart. Section 3 is supported by 63 studies describing the sensory layer—ECG, photoplethysmography, mechanocardiography, laboratory biomarkers and genetic markers—and hybrid analytics, demonstrating that multisensory streams outperform single-modality approaches in predictive accuracy. Section 4 incorporates 59 publications showing how generative models and large medical language models deliver personalized recommendations, round-the-clock monitoring and automation of routine decisions. Section 5 relies on 39 papers that dissect interoperability hurdles, clinical and regulatory validation challenges, privacy concerns and user trust issues. Collectively, the evidence indicates that a multilayer digital cardiovascular twin—comprising sensor data acquisition, hybrid analytics, intelligent agents and a simulation environment—can shift cardiology from reactive treatment to predictive and preventive care; scaling this technology will require coordinated efforts by engineers, clinicians, data scientists, regulators and ethicists to establish interoperable standards, robust privacy safeguards and compelling proof of clinical efficacy.

Artificial intelligence is poised to move cardiology beyond episodic care toward continuous, context-aware management. Next-generation twins will assimilate edge-collected wearables, in-hospital monitors and omics profiles through federated learning, enabling models that learn securely across institutions. Medical LLMs fine-tuned on cardiology corpora will function as conversational co-pilots, translating complex physiological patterns into clinician-ready insights and patient-friendly guidance. Closed-loop AI agents, integrated with implantable and smart drug-delivery systems, promise autonomous titration of therapies and rapid response to arrhythmia or ischemic events.

Realizing this vision will require interoperable data standards, privacy-preserving architectures, scalable cloud-edge hybrids and rigorous multi-center trials with hard cardiovascular endpoints. Regulatory sandboxes should allow iterative validation of adaptive algorithms, while ethical frameworks must address algorithmic bias, data sovereignty and patient consent. Interdisciplinary consortia—uniting engineers, clinicians, data scientists, regulators and ethicists—are essential to translate digital twins into trustworthy, clinically effective tools. If these technical, regulatory and societal gaps are bridged, AI-driven cardiovascular twins could usher in a new era of proactive, personalized heart-health management and substantially reduce the global burden of cardiovascular disease.

## Figures and Tables

**Table 1:** Sensor Devices and Their Roles in Multi-Layered Health Monitoring Architecture.

Device	Sensor Type	Measured Parameters	Connectivity	Edge Capabilities	Role in Architecture	References
Zephyr BioHarness	ECG, Accelerometer	HR, HRV, RR intervals, posture	Bluetooth	RR detection, motion filtering	Wearable + Edge	[23]
Empatica E4	PPG, EDA, Temp, ACC	HR, SpO_2_, GSR, skin temp	Bluetooth, USB	On-device preprocessing	Wearable Layer	[24]
Polar H10	ECG	HR, RR intervals	Bluetooth	Onboard HRV analysis	Sensor + Edge Layer	[24,26]
Xiaomi Smart Band 7	PPG, Accelerometer	HR, SpO_2_, sleep, steps	BLE	Basic HR tracking	Low-power Wearable	[23]
Shimmer3 GSR+	PPG, GSR	HR, GSR, skin conductanc	Bluetooth	Raw signal logging	Research-grae Sensor Layer	[25]
Hexoskin Smart Shirt	ECG, Respiration, ACC	HR, respiration rate, HRV	Bluetooth	On-board buffering	Multimodal Wearable	[27]
Raspberry Pi + MAX30102	PPG	HR, SpO_2_, RR, RMSSD, SDNN	I^2^C, Wi-Fi	Python (3.12)-based HRV computation	Edge + AI Input	[28]
Custom EMG on ESP32	EMG (Digital)	RMS, MAV, ZC, SSC (muscle fatigue)	GPIO, UART	Real-time signal classification	Sensor + Edge Layer	[23,26]
Winsen ZPHS01B	Gas, Temp, Humidity	CO_2_, Temp, RH	UART/I^2^C	Digital signal output	Environmental Layer	[31]
Genomic API	Digital API	SNPs, PRS, gene markers	REST API	Cloud analytics	Genetic Input Layer	[29,30,31]

**Table 3:** Fine-Tuned Deep Learning Models for Cardiovascular Disease Diagnosis.

Model Name	Input Data	Base Architecture	Fine-Tuning Method	Target Disease	Reported Accuracy	Reference(s)
Heart Sense Transformer	ECG Image + IoT Sensor Data	Transformer	Custom ECG dataset fine-tuning	Heart Disease	92.6%	[61]
ECG-DL13	ECG Signal	CNN + Transfer Learning	Small ECG datasets	13 Heart Conditions	94.2%	[62]
EchoMed-CNN	Echocardiogram	CNN	Pretrained CNN layers fine-tuned	Endocarditis	90.4%	[63]
Foundation ECG	Single-lead ECG	Transformer (Echo-FM)	Per clinical label fine- tuning	Multiple CVD conditions	88.9%	[64]
Deep Ensemble ECG	ECG Image	Ensemble CNN models	8 models fine-tuned on private ECG dataset	Arrhythmia and related CVD	95.1%	[65]
IoT-ENN Hybrid	Wearable ECG sensor signals	Epistemic Neural Network	Optimized with Boosted Sooty Tern Algorithm	Cardiac Arrhythmias	91.3%	[66]

**Table 4:** Overview of medical LLMs and generative AI frameworks in healthcare.

Model or Topic	Application Area	Architecture	Notable Features	References
ChatGPT in Science and Healthcare	Clinical communication, research	Transformer (Decoder-only)	Conversational assistant, summarization	[67]
AI/ML in Pathology and Medicine	Pathology, diagnostics, education	General overview (including transformers)	Foundational concepts for generative/nongenerative AI	[68]
LLMs in Ophthalmology	Ophthalmology	BERT, GPT-4, PubMed-BERT, ClinicalBERT	Assessment in exams, clinical notes	[69]
Overview of Generative Models	NLP, vision, general AI	GANs, Autoencoders, Diffusion, Transformer	Technical evolution of generative AI	[70]
Generative AI in Healthcare	Clinical documentation, diagnostics	BioGPT, GatorTronGPT, ClinicalBERT	Multimodal applications, LLM integration	[71]
ICN Conference Papers	Image classification, EEG-based detection	CNN, SVM, Decision Trees	Use of medical datasets like HAM10000	[72]
Multilingual LLMs with LoRA	Chatbot, smart cities	BLOOM-7B1 with LoRA + DeepSpeed	Synthetic dataset creation with prompting	[73]
Medical VQA with Generative Models	Visual Question Answering	Transformer-based generative models	Image-text integration in medical domain	[74,82]
Recent Advances in Medical LLMs	Summarization, clinical assistant	BERT, GPT-3, PaLM, LLaMA	Pretraining on MIMIC-III and other datasets	[75]
Clinical LLMs in Mental Health	Psychiatry, psychotherapy support	ChatGPT, BERT-based	Taxonomy of chatbot evaluation, AI-in-the-loop therapy	[83,84]

## Data Availability

Not applicable.

## Abbreviations

The following abbreviations are used in this manuscript:

AI	Artificial Intelligence
AMI	Ambient Intelligence
AUC	Area Under the Curve
BLE	Bluetooth Low Energy
CNN	Convolutional Neural Network
CT	Computed Tomography
CVD	Cardiovascular Disease
DL	Deep Learning
DLT	Distributed Ledger Technology
ECG	Electrocardiography
EDA	Electrodermal Activity
EHR	Electronic Health Record
EMG	Electromyography
GNN	Graph Neural Network
HR	Heart Rate
HRV	Heart Rate Variability
ICU	Intensive Care Unit
IoMT	Internet of Medical Things
IoT	Internet of Things
LLM	Large Language Model
LoRA	Low-Rank Adaptation
LSTM	Long Short-Term Memory
MAE	Mean Absolute Error
ML	Machine Learning
MRI	Magnetic Resonance Imaging
NLP	Natural Language Processing
PPG	Photoplethysmography
PRS	Polygenic Risk Score
RR	R-R Interval
SDNN	Standard Deviation of NN Intervals
SHAP	Shapley Additive Explanations
SVM	Support Vector Machine
VQA	Visual Question Answering
GDPR	General Data Protection Regulation
HL7	Health Level 7
FHIR	Fast Healthcare Interoperability Resources
